# A Case of Renal Artery Thrombosis With Concurrent Adrenal Hemorrhage in Polycythemia Vera

**DOI:** 10.7759/cureus.68809

**Published:** 2024-09-06

**Authors:** Mohamed Fawzi Mudarres, Bahjat Azrieh

**Affiliations:** 1 Nephrology, University of Iowa Hospitals and Clinics, Iowa, USA

**Keywords:** adrenal hemorrhage, aki, general nephrology dialysis and transplantation, polycythemia rubra vera, renal artery thrombosis, spontaneous adrenal hemorrhage

## Abstract

Polycythemia vera (PV) is a rare myeloproliferative neoplasm characterized by the clonal proliferation of hematopoietic stem cells, leading to an elevated red blood cell mass. This hyperproliferative state increases blood viscosity and predisposes patients to thrombotic events, which are a significant cause of morbidity and mortality in PV. The diagnosis of PV is typically confirmed through elevated hemoglobin or hematocrit levels, low serum erythropoietin, and the presence of the Janus kinase 2 (JAK2) mutation. Common complications include venous and arterial thromboses, hemorrhage, and transformation to myelofibrosis or acute leukemia. A 68-year-old female with a history of PV and chronic kidney disease (CKD) presented with uremic symptoms in the form of malaise and nausea. Laboratory investigations indicated acute kidney injury (AKI) and hyperkalemia. Imaging evaluation of renal US Doppler revealed renal artery thrombosis and an incidental adrenal hemorrhage. The patient was managed with intravenous heparin and did not receive thrombolytics or thrombectomy. Her renal function did not improve, necessitating the initiation of hemodialysis (HD) during hospitalization. Over the course of the next few weeks, her renal parameters improved and she managed to be discharged from dialysis.

The primary goal of this study was to highlight a rare presentation of renal artery thrombosis secondary to polycythemia vera (PV) and discuss the complexities involved in managing the underlying disease and its thrombotic complication, particularly in the presence of concomitant bleeding. Effective management of PV-related thrombosis requires a delicate balance between anticoagulation to prevent further thrombotic events while carefully addressing the risk of hemorrhage.

## Introduction

Polycythemia vera is a type of chronic myeloproliferative neoplasm characterized by the clonal proliferation of myeloid cells. It is primarily an acquired disease caused by a mutation in the Janus kinase 2 (JAK2) gene, a key regulator of blood cell production. This mutation leads to the overproduction of red blood cells, with or without increased levels of white blood cells or platelets. The median age at diagnosis is approximately 60 years [1]. Diagnosis is typically suspected due to high hemoglobin levels and confirmed with a bone marrow biopsy. Most patients are diagnosed incidentally when elevated hemoglobin or hematocrit levels are noted on a complete blood count performed for other reasons. Others present with symptoms of the disease, such as headache, dizziness, visual disturbances, pruritus, early satiety, or complications like thrombosis and bleeding [2]. This hematologic disorder results in hyperviscosity, endothelial dysfunction, and abnormal platelet function, contributing to clot formation and thrombotic events [3]. While venous thrombosis is more commonly recognized, arterial thrombosis, including renal artery thrombosis, is a significant and potentially life-threatening complication of PV [4,5]. Clinical presentations vary widely, from asymptomatic cases to severe abdominal or flank pain, nausea, vomiting, and hypertension, depending on the extent and acuteness of the occlusion. It can lead to renovascular hypertension, chronic kidney disease, and end-stage kidney disease [6].

This study highlights a rare instance of renal artery thrombosis leading to acute kidney injury in a patient with polycythemia vera, underscoring the importance of early recognition and comprehensive management of thrombotic complications in this patient population. Literature on renal artery thrombosis secondary to PV is scarce. A review of similar case reports revealed an 81-year-old patient who presented with gastrointestinal symptoms of abdominal pain and vomiting. A CT angiogram showed extensive intraabdominal arterial and venous thromboses. She had high hemoglobin readings and was evaluated with a bone marrow biopsy, which showed features consistent with polycythemia vera. Another case involved a male in his early 60s presenting with a pulmonary embolism and splenic infarction as rare manifestations of diagnosed PV.

## Case presentation

A 68-year-old female presented to the local hospital emergency department with symptoms of generalized pain, fatigue, and hiccups. Initial laboratory results revealed acute renal injury, characterized by an elevated serum creatinine of 9 mg/dL, blood urea nitrogen (BUN) of 110 mg/dL, and hyperkalemia with a potassium level of 5.9 mEq/L. The patient had a history of chronic kidney disease (CKD), with baseline serum creatinine levels ranging from 2 to 2.5 mg/dL. Her medical history includes polycythemia vera (PV), diagnosed in October 2021 with a JAK2 mutation, for which she had been off treatment, including aspirin and hydroxyurea, for two years. Upon admission, laboratory values are noted below (Table 1).

**Table 1:** Initial lab results at admission.

Labs	Results	Reference range
Sodium	127 mEq/L	135-145 mEq/L
Potassium	6.6 mEq/L	3.5-5.0 mEq/L
Chloride	88 mEq/L	95-107 mEq/L
CO_2_	16 mEq/L	22-29 mEq/L
Anion gap	23 mEq/L	<17 mEq/L
Blood urea nitrogen (BUN)	105 mg/dL	10-20 mg/dL
Creatinine	8.37 mg/dL	0.51-0.95 mg/dL
eGFRcr (CKD-EPI 2021)	5 mL/min/1.73 m²	>90 mL/min/1.73 m²
Glucose	109 mg/dL	65-139 mg/dL
Calcium	8.3 mg/dL	8.5-10.5 mg/dL
Magnesium	2.1 mg/dL	1.5-2.9 mg/dL
Phosphorus	4.7 mg/dL	2.5-4.5 mg/dL
WBC count	25.5 K/µL	3.7-10.5 K/µL
Hemoglobin	19.0 g/dL	11.9-15.5 g/dL
Hematocrit	61%	35-47%
Platelet count	175 K/µL	150-400 K/µL

The patient also had a history of deep vein thrombosis (DVT) of the upper extremities, diagnosed in 2020, and was on apixaban 5 mg twice daily. A CT pulmonary angiogram performed at an outside hospital before her transfer to our facility did not show signs of pulmonary embolism but did reveal bilateral adrenal hemorrhage. Additionally, a CT head scan showed no evidence of stroke. The patient was transferred to our hospital for potential hemodialysis, given her impaired renal function.

**Figure 1:**
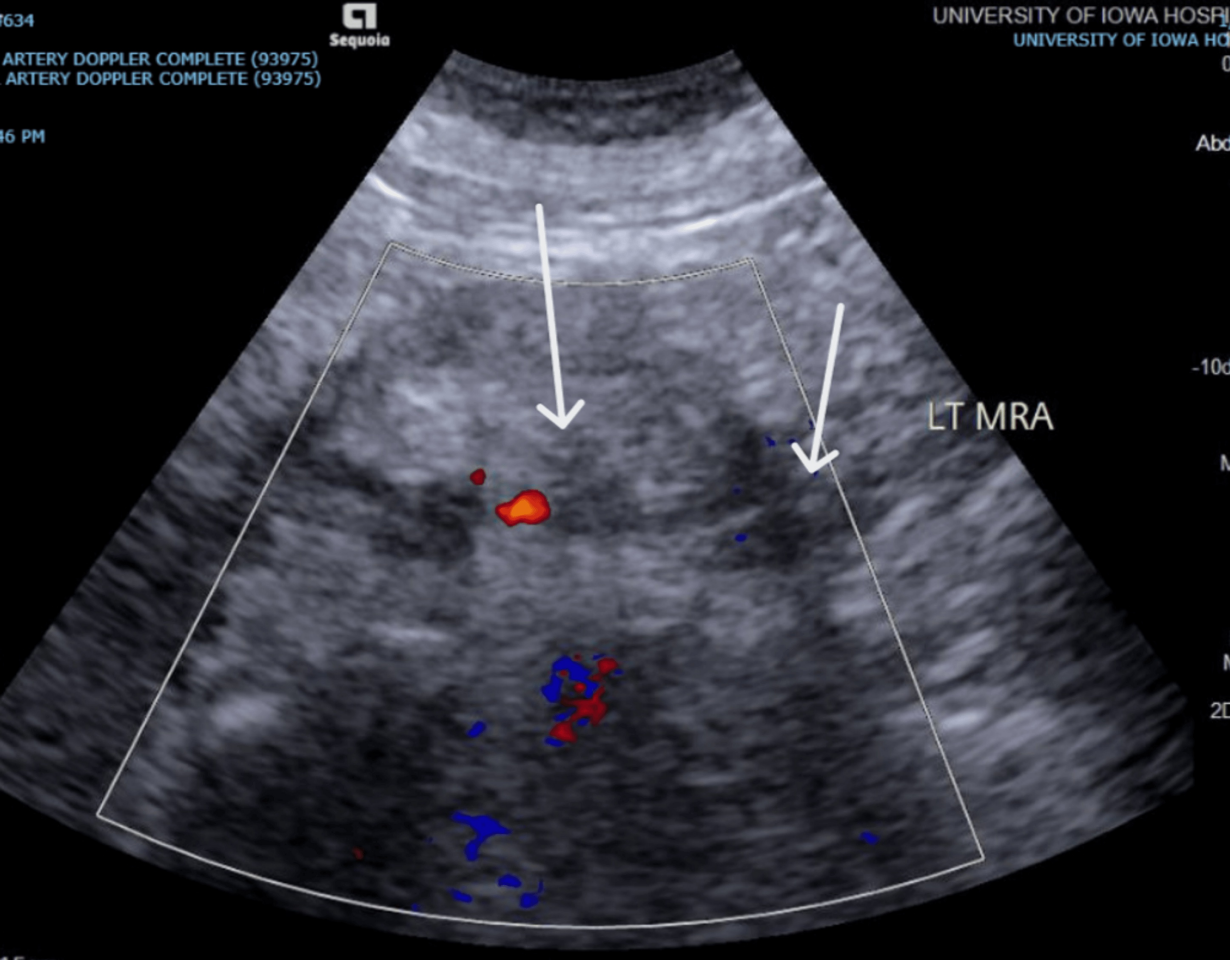
Doppler ultrasound of the renal arteries. Absent color flow signals within the left renal artery (arrows).

The workup of her acute kidney injury (AKI) included a Doppler ultrasound of the renal arteries, which demonstrated absent Doppler waveforms in the left kidney, suggestive of vascular occlusion and indicative of renal artery thrombosis (Figure 1). The AKI was non-oliguric, with a urine output of approximately 1 L per day. Admission urinalysis was positive for +1 blood and +2 protein, with microscopy negative for pyuria and hematuria. Her urine protein creatinine ratio was abnormally high (Table 2).

**Table 2:** Urine examination results of the patient.

Urine studies	Results	Reference range
Color	Yellow	Straw, yellow
Clarity	Clear	Clear
Blood	2+	Negative
Protein	2+	Negative
WBC	2	0-5/HPF
RBC	23	0-2/HPF
Protein/creatinine ratio	1.74	≤0.20

**Figure 2:**
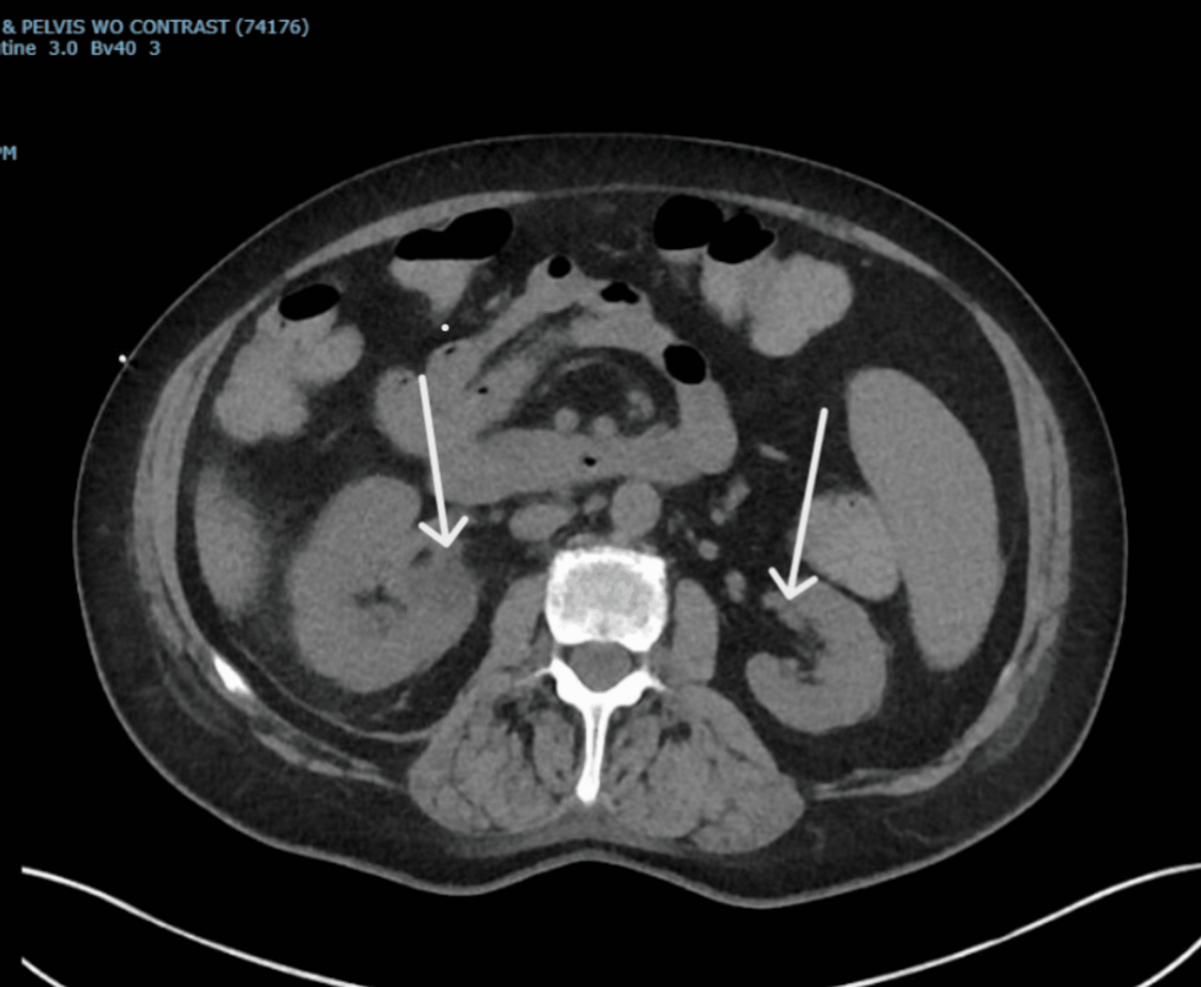
CT scan of the abdomen and pelvis without contrast. Bilateral adrenal enlargement, more pronounced on the right side, showing high attenuation areas (hyperdense) consistent with an adrenal hematoma (arrows).

**Figure 3:**
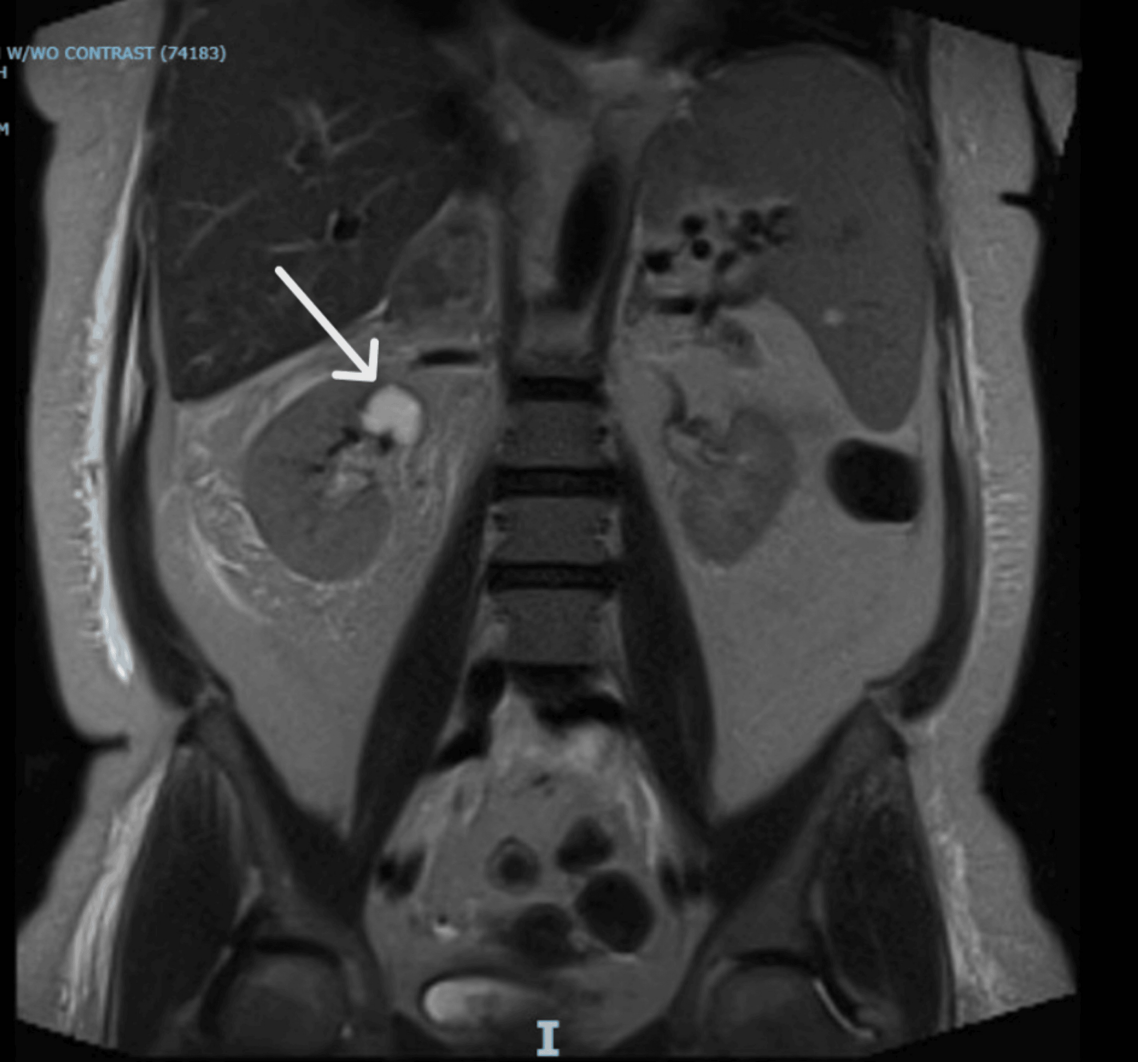
MRI abdomen without contrast. A well-defined mass within the right adrenal gland, which is hyperintense on T1-weighted images, is consistent with a hematoma (arrow).

The patient underwent emergent therapeutic phlebotomy due to high hematocrit levels, removing 500 mL of blood. A CT scan of the abdomen and pelvis without contrast, performed two days post-admission, showed enlarged bilateral adrenal glands, more pronounced on the right, with internal hyperdense components suggestive of stable bilateral adrenal hemorrhage compared to the initial imaging (Figure 2). Further characterization by MRI of the abdomen the following day revealed a rounded right adrenal mass, likely representing a hematoma, with no underlying enhancing components to suggest malignancy (Figure 3).

The nephrology team evaluated the patient and concluded that her AKI was at least partially caused by renal artery thrombosis superimposed on CKD stage IIIa. The interventional radiology (IR) team recommended thrombolysis; however, due to the high risk of bleeding, no intervention was performed. Following discussions with the hematology team, a heparin drip was initiated under a high-risk protocol, and the patient was later transitioned to apixaban one week later.

Due to worsening renal parameters, a tunneled catheter was placed on day 10 of admission, and dialysis was initiated the following day. The patient became oliguric and exhibited slowly progressive cognitive symptoms, including forgetfulness and disorientation. Dialysis continued for a week before the permcath was removed due to bacteremia, with blood cultures positive for *Staphylococcus haemolyticus* and *Enterococcus faecium*. She completed a course of daptomycin as an inpatient. Fortunately, no further dialysis was required due to improvement in renal function and urine output.

The patient was discharged after one month, with serum creatinine levels decreasing to the mid 2 mg/dL range, correlating with her baseline. Electrolyte imbalances, including hyperkalemia and metabolic acidosis, were corrected. She was prescribed hydroxyurea 500 mg daily for her PV and arranged to follow-up with hematology as an outpatient.

## Discussion

Polycythemia vera (PV) is a chronic myeloproliferative neoplasm (MPN) characterized by an elevated red blood cell mass, often accompanied by increased platelet and white blood cell counts. Before confirming a diagnosis of PV, it is essential to rule out other conditions, such as chronic hypoxia and erythropoietin-secreting tumors [2].

PV can manifest at any age, although the median age at diagnosis is approximately 60 years. Patients are often diagnosed incidentally during routine blood tests that reveal elevated hemoglobin or hematocrit levels. Symptomatic patients may present with headaches, dizziness, visual disturbances, or pruritus. Complications like thrombosis or bleeding may also be initial indicators. Additionally, PV patients have an increased risk of leukemic transformation and myelofibrosis [4].

The World Health Organization (WHO) analysis of over 1,500 patients revealed a median hemoglobin level of 18.4 g/dL and a median hematocrit of 55%. Hypertension was present in approximately 46% of patients, with thrombosis and bleeding incidences of 23% and 4%, respectively [4]. A common symptom of PV is pruritus, often triggered by water exposure (aquagenic pruritus). The exact cause of pruritus in PV is unclear, but theories include mast cell degranulation and the release of histamine, fibrinolytic factors, prostaglandins, interleukin-31, and adenosine diphosphate from red cells. Aspirin has been found to relieve pruritus in several patients, supporting the involvement of prostaglandins [7].

Erythromelalgia, characterized by burning pain in the distal extremities accompanied by erythema, pallor, or cyanosis, is considered pathognomonic for microvascular thrombotic complications in PV [4]. Patients with PV are also at increased risk for various thrombotic events, such as cerebrovascular accidents, myocardial infarctions, superficial thrombophlebitis, deep vein thrombosis, and pulmonary embolism, as well as hemorrhage. This hypercoagulable state is attributed to abnormalities in blood viscosity, platelets, and leukocytes [4].

Other symptoms associated with PV include transient visual disturbances, such as transient blindness or scotomas, and gastrointestinal symptoms resulting from altered gastric mucosal blood flow due to increased blood viscosity and histamine release from tissue basophils [8,9].

The core laboratory findings for PV include elevated hemoglobin or hematocrit levels. For diagnosis, males should have a hemoglobin level of at least 16.5 g/dL or a hematocrit level of ≥49%, and females should have a hemoglobin level of at least 16 g/dL or a hematocrit level of ≥48%. Thrombocytosis and leukocytosis are also common. However, the total white blood cell count may not accurately reflect disease activity as the increase is predominantly in neutrophils rather than lymphocytes or monocytes [10]. Peripheral smear analysis correlates with these findings in the pre-polycythemic and overt-polycythemic stages. In the post-polycythemic myelofibrosis stage, a leukoerythroblastic picture with teardrop-shaped red blood cells and circulating nucleated red cells is observed [10].

Bone marrow aspiration and biopsy typically reveal hypercellularity for age with trilineage growth, including prominent erythroid, granulocytic, and megakaryocytic proliferation. In the post-polycythemic myelofibrosis stage, bone marrow findings include cytopenias, ineffective erythropoiesis, fibrosis, and extramedullary hematopoiesis [4,10].

Genetic screening from peripheral blood shows a high sensitivity (95%-100%) for JAK2 (Janus kinase 2) mutations involving either exon 14 or 12, which helps distinguish PV from secondary polycythemia [11]. While the JAK2 mutation is not exclusive to PV, it is also present in a significant proportion of patients with essential thrombocythemia and primary myelofibrosis. PV patients typically have low serum erythropoietin (EPO) concentrations, and low EPO levels are highly specific for PV, with a specificity of 98%. Elevated EPO levels are unusual in PV and suggest secondary erythrocytosis [12].

The cornerstone of PV treatment includes controlling the red blood cell (RBC) mass, managing symptoms, and preventing PV-associated complications [4]. The recommended target for hematocrit is <45%, typically achieved initially through periodic therapeutic phlebotomy. For high-risk patients - those older than 60 years or with a history of thrombosis - cytoreductive therapy is used alongside phlebotomy. Hydroxyurea is the first-line cytoreductive agent for non-pregnant patients, while interferon-alpha (IFNa) is an alternative option [9]. A low-dose aspirin regimen is recommended for all PV patients due to its efficacy in alleviating microvascular symptoms and reducing mortality from cardiovascular and thromboembolic events [13].

Renal infarction, a form of arterial thrombosis in PV, can also have other etiologies, such as cardioembolic disease, renal artery injury (commonly due to dissection), and other hypercoagulable states [14]. Although renal infarction is typically unilateral, bilateral involvement has been reported in nearly 20% of cases [14,15].

Patients with acute renal infarction usually present with sudden onset flank or abdominal pain, which may be accompanied by nausea, vomiting, and occasionally fever [16]. Hematuria occurs in about one-third of cases, and rising serum creatinine levels are especially notable in patients with bilateral or large unilateral emboli [16].

Diagnosis of renal infarction is primarily achieved through contrast-enhanced imaging, typically a contrast-enhanced CT scan, which reveals a wedge-shaped perfusion defect or, in complete central renal artery occlusion cases, a non-enhancing kidney [14,17,18].

The initial management of renal infarction focuses on rapidly identifying patients who may benefit from revascularization. Candidates include those with arterial dissection, complete or partial renal artery occlusion, or major segmental artery involvement occurring within 24 hours. Those with significant acute kidney injury (AKI), new or worsening hypertension, or persistent symptoms beyond 24 hours may also be considered for intervention [14].

In cases where revascularization is not pursued, systemic anticoagulation remains the mainstay of treatment, particularly for patients with thromboembolic risk factors. Concurrent antiplatelet therapy may be administered to those undergoing angioplasty or stent placement or with underlying vascular abnormalities. In our case, the patient was already on aspirin as part of PV management [14-16]. However, due to the presence of an adrenal hematoma and the high risk of bleeding, intervention was not pursued. After a thorough discussion with a multidisciplinary team, it was determined that the potential complications of revascularization outweighed the benefits.

## Conclusions

This case of a 68-year-old female with a complex medical history, including polycythemia vera (PV) and deep vein thrombosis, presenting with renal artery thrombosis, acute kidney injury, and bilateral adrenal hemorrhage, poses a management dilemma. It also illustrates the importance of a multidisciplinary approach in the management of PV that is complicated by thrombosis and bleeding and the need for vigilant monitoring and adaptation of treatment strategies to mitigate the risks of disease progression and associated morbidities.
